# Treatment of multiple vulvar angiokeratomas using long-pulsed 1064-nm Nd:Yag laser: a case report

**DOI:** 10.1093/jscr/rjae740

**Published:** 2024-11-24

**Authors:** Fabrizio Rocco Mancuso, Emanuele Maria Cipollini, Tiziano Zingoni, Irene Fusco, Mario Sannino, Giovanni Cannarozzo

**Affiliations:** Dermatology Unit, Health Sciences Department, University of Florence, Viale GB Morgagni, 48, 50134, Florence, Italy; Dermatology Unit, Health Sciences Department, University of Florence, Viale GB Morgagni, 48, 50134, Florence, Italy; El.En. Group, Department of Clinical Research and Practice, Via Baldanzese 17, 50041 Calenzano, Italy; El.En. Group, Department of Clinical Research and Practice, Via Baldanzese 17, 50041 Calenzano, Italy; Laser in Dermatology Unit, University of Rome Tor Vergata, Via Cracovia 50, 00133, Rome, Italy; Laser in Dermatology Unit, University of Rome Tor Vergata, Via Cracovia 50, 00133, Rome, Italy

**Keywords:** vulvar angiokeratoma, angiokeratoma of Fordyce, laser treatment, Nd:Yag laser

## Abstract

The purpose of this clinical case was to test the efficacy and safety of the long-pulsed 1064-nm neodymium-YAG laser in the treatment of multiple angiokeratomas on the labia majora of the vulva. Benign vascular neoplasms known as angiokeratomas are characterized by well-defined, hyperkeratotic, reddish-black papules, or plaques. The patient underwent one treatment session with a long-pulsed 1064-nm neodymium-YAG laser. A clinical photographic assessment was performed immediately after the laser session and at 14 days follow-up. At the follow-up appointment, the patient reported no significant discomfort and physical examination revealed complete resolution of all angiokeratomas. The skin of the labia majora appeared healthy, with no visible scarring or pigmentation changes. The patient expressed high satisfaction with the cosmetic outcome. This case supports the use of laser systems for managing angiokeratomas in delicate areas like the genital region, providing significant aesthetic improvement and patient satisfaction.

## Introduction

Angiokeratomas, also known as angiokeratoma of Fordyce, are asymptomatic, dark red, purple, blue, or black, smooth-surfaced, dome-shaped, benign papules. They typically measure from 2 to 8 mm and consist of dilated superficial blood vessels with hyperkeratosis of the overlying epidermis. Although generally benign, bleeding may occur secondary to trauma. These lesions can be solitary or multiple, often developing between the ages of 20–50 years and increasing in number during pregnancy [1, 2].

## Case presentation

A 50-year-old woman presented with multiple angiokeratomas on the labia majora of the vulva. She reported that these lesions had been present since childhood but had become more prominent and numerous over the years. The patient expressed concern about the appearance and occasional bleeding of the lesions. Her medical history showed no significant findings, which helped rule out potential genodermatoses such as Fabry disease [3].

**Figure 1:**
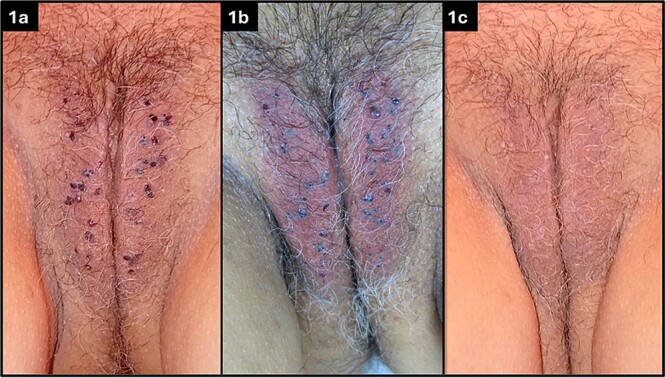
Multiple angiokeratomas on the labia majora of the vulva (a), immediately following the procedure (b), and at 14 days follow-up visit (c).

On physical examination, numerous small, reddish-purple papules were noted on the labia majora (Fig. 1a). Dermoscopy confirmed the diagnosis of angiokeratomas, showing multiple lacunae with a well-demarcated vascular pattern [4].

The patient underwent treatment with a long-pulsed 1064-nm neodymium-YAG laser. The laser was set to dual-pulse mode (5–15 ms) with an interval of 20 ms between them and fluence of 90–95 J/cm^2^. A 5-mm spot size handpiece with an integrated cooling system was used to minimize discomfort during the treatment.

Local anaesthesia was not administered, and the treatment was well tolerated by the patient, who reported only mild pain and burning sensations. Each lesion was carefully targeted to ensure effective treatment while minimizing damage to surrounding tissue. The session lasted ~20 minutes.

Immediately after the laser treatment, the colour of the angiokeratomas changed from reddish-purple to greyish, with surrounding erythema (Fig. 1b). To improve post-treatment tolerance and minimize potential adverse effects, cold saline-soaked gauzes and air cooling were applied to the treated area for about 10 minutes.

The patient was advised on post-treatment care, including avoiding friction and trauma to the treated area and applying a topical antibiotic ointment. She was scheduled for a follow-up visit 14 days after the procedure.

At the follow-up appointment, the patient reported no significant discomfort. Physical examination revealed complete resolution of all angiokeratomas (Fig. 1c). The skin of the labia majora appeared healthy, with no visible scarring or pigmentation changes. The patient expressed high satisfaction with the cosmetic outcome.

## Discussion

This case demonstrates the efficacy and safety of using the long-pulsed 1064-nm neodymium-YAG laser in treating angiokeratomas of the vulva. The dual-pulse mode and 1064 nm wavelength effectively target and coagulate the blood vessels within the lesions, resulting in their resolution. The immediate colour change observed post-treatment is indicative of successful vessel coagulation.

Previous literature supports the use of the long-pulsed 1064-nm neodymium-YAG laser for various vascular lesions, though specific reports on vulvar angiokeratomas are limited. This case contributes valuable evidence for the laser’s application in such sensitive areas, showing excellent aesthetic results without adverse effects [5–7].

The treatment of vulvar angiokeratomas with a long-pulsed 1064-nm neodymium-YAG laser in dual-pulse mode in a single session was highly effective and safe. The lesions resolved completely within 14 days, with no complications or scarring. This case supports the use of laser systems for managing angiokeratomas in delicate areas like the genital region, providing significant aesthetic improvement and patient satisfaction. Further studies are warranted to establish standardized protocols and confirm long-term outcomes.

## Data Availability

On reasonable request, the corresponding author will provide the information supporting the study’s results.
